# Radiation-Induced Peripheral Malignant Nerve Sheath Tumor Arising from Vestibular Schwannoma after Linac-Based Stereotactic Radiation Therapy: A Case Report and Review of Literatures

**DOI:** 10.1155/2012/648191

**Published:** 2012-07-09

**Authors:** Putipun Puataweepong, Taweesak Janwityanujit, Noppadol Larbcharoensub, Mantana Dhanachai

**Affiliations:** ^1^Department of Radiology, Faculty of Medicine, Ramathibodi Hospital, Mahidol University, Bangkok 10400, Thailand; ^2^Department of Surgery, Faculty of Medicine, Ramathibodi Hospital, Mahidol University, Bangkok 10400, Thailand; ^3^Department of Pathology, Faculty of Medicine, Ramathibodi Hospital, Mahidol University, Bangkok 10400, Thailand

## Abstract

In recent years the use of stereotactic radiation for vestibular schwannomas has increased worldwide. However, malignant transformation associated with radiation, although uncommon, has been reported in recent publications. We present a case of the 34 year-old female who had left vestibular schwannoma and who underwent surgery and postoperative stereotactic radiotherapy (SRT), hypofraction in 2005. At 6 years after SRT, the patient came with left facial palsy and severe headache. CT brain revealed progression in size with cystic and hemorrhagic changes of the preexisting tumor at left CPA with new obstructive hydrocephalus. Partial tumor removal was done, and the pathological report was malignant peripheral nerve sheath tumor (MPNST). Regarding the uncertainty of carcinogenesis risk, we should still practice radiation therapy with caution, especially in the young patient with tumor predisposition syndrome. Because of low incidence of MPNST after radiation, it should not be a major decision about giving radiotherapy. However, with the poor prognosis of MPNST, this possibility should be explained to the patient before radiation treatment option.

## 1. Introduction

In recent years, stereotactic radiosurgery (SRS) and fractionated stereotactic radiotherapy (SRT) have been considered the best management strategy for the majority of small- to-medium size vestibular schwannomas (VS) [1, 2]. The widespread use of SRS/SRT in benign VS causes a great concern regarding radiation-induced malignancy, which is the rare but serious complication after radiation. 

We reported a case with sporadic benign VS, which had transformed to malignant peripheral nerve sheath tumor (MPNST) after 6 years of SRT with a review of the literature.

## 2. Case Report

**Figure 1:**
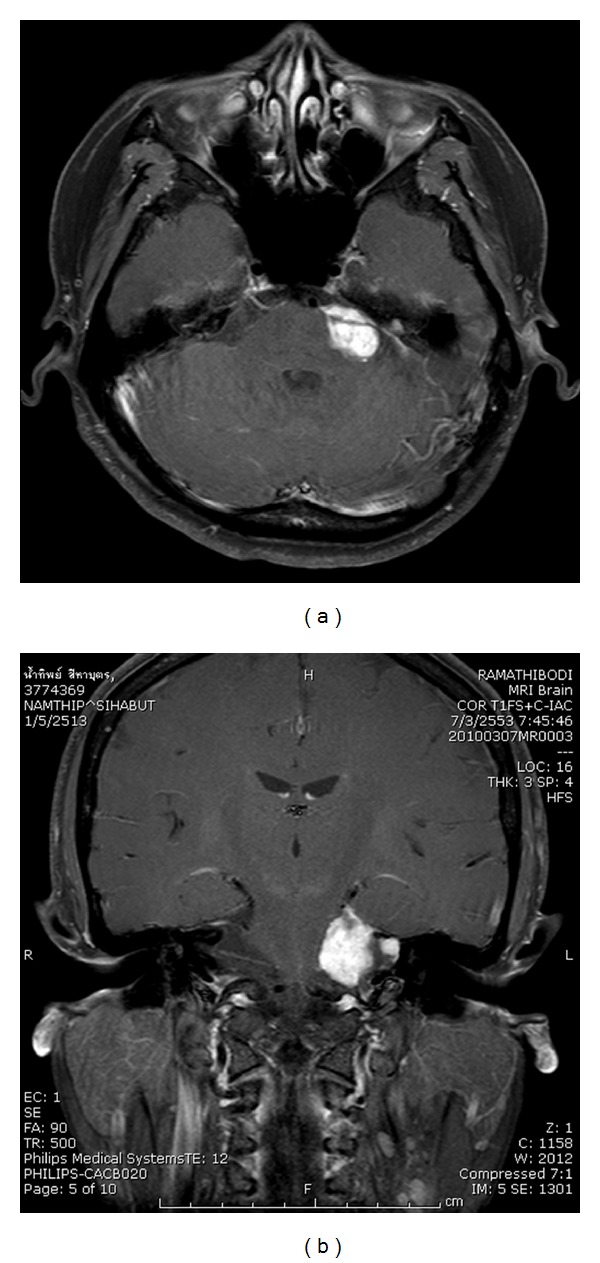
Axial and coronal T1W MRI with contrast showing a left vestibular schwannoma at the time before SRT (2005).

A 34-year-old female, without a familial history of neurofibromatosis, presented with left hearing disturbance. Magnetic resonance imaging (MRI) in 2004 showed VS at left cerebellopontine angle. Two times of left retrosigmoid approach with partial tumor removal were done during 2004. Pathological report was schwannoma WHO grade I. At 1 year after the second surgery, SRT, hypofraction was given due to regrowth of the tumor. Linear accelerator base system (6 MV dedicated Linac, Varian; with X-knife planning system version 3&4, Radionics) was used for SRT treatment. Four isocenters with total average dose of 30 Gray in 6 fractions prescribed at 80% isodose line were given to the patient during 2 weeks period. The tumor volume was 4.8 cc. The tumor had been well controlled for 6 years after SRT.  Figure 1 revealed the MRI Axial and Coronal showing a left VS at the time before SRT.

**Figure 2:**
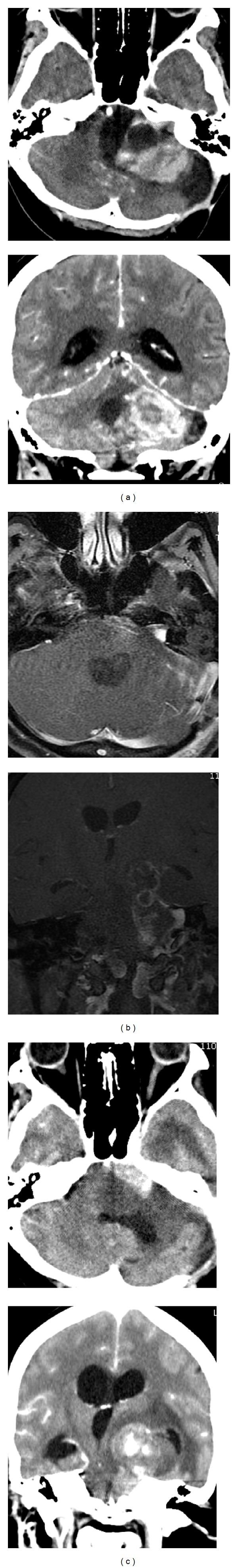
(a) Axial and Coronal CT with contrast showing radiological recurrence consistent with symptomatic recurrence 6 years after SRT (2011). (b) MRI was done at 3 weeks after recraniotomy with partial tumor removal. There was residual tumor with intense enhancement at the left internal auditory canal, measured about 0.9 × 0.8 × 1.5 cm^3^ in size, tumor volume measured about 0.535 cc. Newly seen was the cluster of hypointense T1/heterogeneous dark-hypointense T2 lesions with hyperintense T1 rim at the superior aspect of the surgical site, involving the left-sided prepontine cistern left-sided perimesencephalic and ambient cistern measured about 2.6 × 1.6 × 2.4 cm in AP, transverse and vertical greatest dimension. These findings were suggestive of the resolving hematomas. (c) The last CT scan of the patient. During 10 fractions of 3D-CRT, the patient developed deterioration of consciousness. CT scan showed rapid progression of residual tumor with internal bleeding. Obstructive hydrocephalus was more severe.

The patient presented with left facial palsy, dysphagia, and right hemiparesis 6 years after SRT. Computer tomography revealed marked progression in size with cystic and hemorrhagic changes of the preexisting tumor at left CPA with new obstructive hydrocephalus (Figure 2(a)). Craniotomy with tumor removal was done immediately. MRI was performed at 3 weeks after operation, and the study shows small residual tumor at the left internal auditory canal with the cluster lesions at the superior aspect of the surgical site that is compatible with the resolving hematomas (Figure 2(b)). The patient had improvement of her neurological symptom after surgery. The pathological report was malignant peripheral nerve sheath tumor arising on schwannoma, with heterogenous mesenchymal rhabdomyoblastic and chondroblastic differentiation, WHO grade IV. 

Because of malignant histology and small residual tumor, the patient underwent postoperative radiation therapy with 3-dimensional conformal radiation therapy (3D-CRT). A total dose of 54 Gray in 27 fractions was planned for the patient. After 20 Gray in 10 fractions of 3D-CRT, the patient developed deterioration of consciousness. CT emergency was done and showed progression of residual tumor with internal bleeding (Figure 2(c)). Obstructive hydrocephalus was more severe. Ventriculoperitoneal shunt was performed immediately. After VP shunt, the patient still had no cooperation and showed no response to deep pain. Because the prognosis of the patient was poor, we discussed with her husband about the end of life care. Finally, the patient was discharged home for best supportive care 1 month later.

### 2.1. Histology

**Figure 3:**
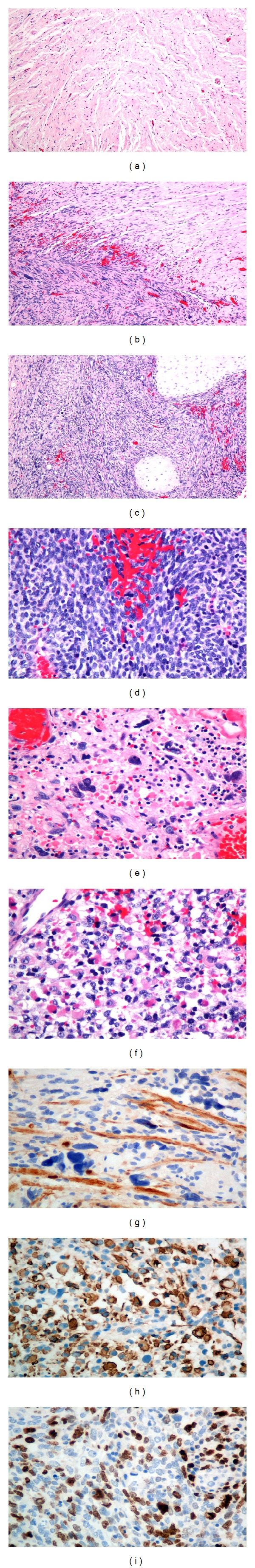
Histopathology of the first diagnosis of schwannoma (1(a), ×40), histopathology of the following specimen showing malignant peripheral nerve sheath tumor (MPNST) arising in schwannoma (1(b), ×40), MPNST with chondrosarcomatous component (1(c), ×40), MPNST with area composed of round cell tumor and brisk mitotic activity (1(d), ×100), MPNST with pleomorphic nuclei (1(e), ×400), MPNST with rhabdomyoblastic differentiation (1(f), ×400), MPNST showing immunonegativity for S-100 (1(g), X400), MPNST showing sarcomeric actin immunoreactivity (1(h), ×400), and MPNST with high Ki-67 immunohistochemistry (1(i), ×400).

The two pathological specimens were examined in two occasions as shown in Figure 3. The first specimen reveals low cellularity spindle cell tumor with minimal nuclear atypia and fibrillary cytoplasm that are compatible with schwannoma (Figure 3(a)). 

 The second specimen was performed after irradiation and composed of markedly hypercellularity and hyperchromatic spindle cells growing in fasciculated pattern. The cytoplasm is typically light staining and indistinct (Figure 3(b)). A few areas of markedly pleomorphic nuclei, brisk mitotic activity, and round cells epithelioid appearance are also seen (Figures 3(d) and 3(e)).

 The capacity of MPNST to undergo focal mesenchymal differentiation is well known. Rhabdomyosarcoma is most frequent. The other sarcomatous component may be present such as chondrosarcoma and osteosarcoma. In this case, pluridirectional differentiations are observed as chondrosarcomatous component (Figure 3(c)) and rhabdomyoblastic component (Figure 3(f)).

 Immunohistochemically, S-100 is negative (Figure 3(g)) and confirms rhabdoid/skeletal muscle differentiation with positive staining with sarcomeric actin (Figure 3(h)). The high proliferative index (Ki 67) is also seen supporting malignant behavior (Figure 3(i)).

## 3. Discussion 

Stereotactic radiosurgery (SRS) or fractionated stereotactic radiotherapy (SRT) is techniques to administer precisely directed, high-dose irradiation that tightly conforms to an intracranial target to create a desired radiobiologic response while minimizing radiation dose to the surrounding normal tissues. In recent years, SRS/SRT has become a commonly used modality for brain tumor especially in VS treatment. Although radiation-induced malignant brain tumors possibly from this technique have been reported, there were only few studies with complete review. The purpose of our study is, therefore, to present another case reports in the radiation-induced MPNST with an extensive review of literatures.

In 1948, Cahan et al. [22] established the criteria of a radiation-induced tumor which included (1) a second tumor occurs within the radiation field, and it was not present at the time of radiation, (2) a latency period is required between radiotherapy and tumor development (several years), (3) a histological difference must exist between the primary and the new tumor, and (4) the patient should not have any genetic predisposition for cancer development. In this report, it seems that our case might not fulfill all of the Cahan's criteria by some reasons. Firstly, a primary and the new tumor do not have a completely histological difference. Secondly, there was only 1 mL of tumor tissue from ultrasonic aspirator sent for pathological section which may not sufficiently represent the rest of tumor. Thirdly, we found that there were some areas of increased vascularity and adhesion to the upper brainstem causing difficulty in tumor removal during the first two operations which may be signs of a higher than grade 1 tumor. However, we believe that it is still reasonable to consider or suspect that radiation effects from SRS/SRT have had a major role in the pathogenesis of a malignant conversion from the primary benign tumor into MPNST in this patient. 

The incidence of MPNST is very rare, approximately 0.001% [23]. Most cases developed sporadically (50%), 30% arising from malignant transformation of benign schwannoma, and radiation is a well-established carcinogen and associated with malignant transformation of vestibular schwannoma (VS). Comey et al. [11] described that, after radiation, most of the irradiated cells usually undergo cytoplasmic vacuolization and subsequent cell death, Rarely, some of the surviving cells might acquire genetic mutations, which are responsible for the malignant transformation of VS. From literature reviews, we found 24 [3–21] (including our report) cases of malignant brain tumor following stereotactic radiotherapy of VS (Table 1). There was 16 cases of malignant transformation of VS. De novo secondary tumor (the new tumor that appeared in the radiation field) was found in 6 cases, and 2 patients had rapid growth of tumor several years after radiation. Most of them received SRS and had histological proof of a malignant tumor after radiation (20 cases). Twelve cases were neurofibromatosis 2 (NF-2). The median time to develop secondary tumor was 6 years. 

NF-2 is a rare (1 : 40,000) autosomal dominant disease that is caused by mutation of the NF2 tumor suppressor gene. From our reviews, we found a high incidence of malignant transformation in irradiated NF-2 patients (52%). Baser et al. [4] surveyed 1348 NF-2 patients and reported that the incidence of malignant transformation was 10% in the irradiated patient compared to 0.7% in the nonirradiated. They concluded that NF-2 patients had a 14-fold increased risk of developing malignant brain tumor after radiation. Regarding the higher incidence of radiation-induced malignant transformation, observation and surgery are the preferred treatment option for NF-2 patients, particularly for those who are young. 

After radiation exposure, there are two possible outcomes. One is the deterministic effect, which has a threshold of dose and a severity of the effects which are dose related. Another effect is called stochastic, which is carcinogenesis, hereditary effects fall in this category. If somatic cells are exposed to radiation, the probability of cancer increases with dose, probably with no threshold. But the severity of the cancer is not dose related. A cancer induced by 1 Gray is no worse than one induced by 0.1 Gray. But of course the probability of its induction is increased. An advanced conformal radiation technique, such as 3D-CRT, IMRT or SRS/SRT, can deliver high radiation dose to tumor with lower dose to normal tissue. This might reduce only the deterministic effect, but the effect to the stochastic is still unknown. Regarding the uncertainty of carcinogenesis risk, we should still practice radiation therapy with caution, especially in young patients with tumor predisposition syndrome. Because of low incidence of MPNST after radiation, it should not be a major decision about giving radiotherapy. However, with the poor prognosis of MPNST, this possibility should be explained to the patient before radiation treatment option.

## Figures and Tables

**Table 1:** The cases of malignant transformation of VS after stereotactic radiation.

Author	Age/sex	NF2	RT tech	Final pathology	Years to 2nd tumor
Thomsen et al., 2000 [3]	19/F	Y	SRS	Sarcoma	6
Baser et al., 2000 [4]	N/A	Y	SRS	3 MPNSTs 1 malignant meningioma, 1 malignant ependymoma (de novo)	N/A
Ho and Kveton, 2002 [5]	14/F	Y	SRS	Rapid growth (no patho)	7 months
McEvoy and Kitchen, 2003 [6]	22/M	Y	SRS	Rapid growth (no patho)	2
Bari et al., 2002 [7]	28/F	Y	SRS	MPNST	4
Rowe et al., 2008 [8]	F	Y	SRS	Malignant glioma (de novo)	3
Carlson et al., 2010 [9]	25/F	Y	SRT	Rhabdosarcoma (de novo)	10
Husseini et al., 2011 [10]	20/M	Y	SRS	MPNST	5
Comey et al., 1998 [11]	50/M	N	SRS	Triton	5
Shamisa et al., 2001 [12]	57/F	N	SRS	GBM (de novo)	7.5
Hanabusa et al., 2001 [13]	51/F	N	SRS	Sarcoma	6 months
Shin et al., 2002 [14]	26/F	N	SRS	MPNST	6
Wilkinson et al., 2004 [15]	53/M	N	SRS	MPNST	4
Muracciole et al., 2004 [16]	61/F	N	SRS	Triton	6
Maire et al., 2006 [17]	N/A	N	SRT	MPNST	19
Balasubramaniam et al., 2007 [18]	64/F	N	SRT	GBM (de novo)	5
Yang et al., 2010 [19]	74/M	N	SRS	Sarcoma	6
Demetriades et al., 2010 [20]	37/M	N	SRS	MPNST	10
Akamatsu et al., 2010 [21]	67/F	N	SRS	MPNST	7.5
Our case 2011	34/F	N	SRT	MPNST	6
